# Not Just Being Lifted: Infants are Sensitive to Delay During a Pick-Up Routine

**DOI:** 10.3389/fpsyg.2015.02065

**Published:** 2016-01-20

**Authors:** Valentina Fantasia, Gabriela Markova, Alessandra Fasulo, Alan Costall, Vasudevi Reddy

**Affiliations:** ^1^Naturalistic Social Cognition, Max Planck Institute for Human DevelopmentBerlin, Germany; ^2^Faculty of Psychology, University of ViennaVienna, Austria; ^3^Department of Psychology, University of PortsmouthPortsmouth, UK

**Keywords:** pick-up, early routines, body tension, violations, cooperation, action understanding

## Abstract

In the present study we observed whether infants show online adjustments to the mother’s incipient action by looking at their sensitivity to changes as the pick-up unfolded. Twenty-three 3-month-old infants and their mothers were observed in the lab, where mothers were instructed (1) to pick-up their infants as they usually did (normal pick-up), and then (2) to delay the pick-up for 6 s after placing their hands on the infants’ waist (delayed pick-up). In both Normal and Delayed conditions infant’s body tension, affective displays and gaze shifts were coded during three phases: Approach, Contact, and Lift. Additionally, a measure of infants’ head support in terms of head lag at the beginning and end of Lift was computed. Results showed that during normal pick-up infants tensed up their body during the Approach phase and increased their tension during contact, maintaining it through Lift; their head was also supported and in line with their body during Lift. When the pick-up was delayed, infants also tensed their body during Approach, yet this tension did not increase during the Contact phase and was significantly lower at Lift. Their head support was also lower in the Delayed condition and they shifted their gazes away from their mothers’ face more often than in the Normal condition. These results suggest that infants are sensitive to changes of the timing of the pick-up sequence, which in turn may have affected their contribution to the interaction.

## Introduction

From their very 1st days of life, infants are involved in sequential and repeated activities or routines, such as having a diaper changed (Nomikou and Rohlfing, 2011), being fed (Kochukhova and Gredebäck, 2010), playing social games (Ratner and Bruner, 1978; Bruner and Sherwood, 1983; Fantasia et al., 2014b), or being picked up (Service, 1984; Lamb and Malkin, 1986; Reddy et al., 2013). Because of their predictability, these routines support infants’ ability to understand and take part in others’ goal-directed actions, for instance by learning to anticipate the caregivers’ behavior (Gredebäck and Melinder, 2010) and respond to affective and interactive temporal contingencies (Gratier, 2003; Hilbrink et al., 2015). The goal of the present study was to examine infants’ contribution to the caregiver’s pick-up behaviors by studying their sensitivity to changes in the pick-up timing.

### Routines as Contexts of Co-Operation

Being involved in and directly addressed as recipients of others’ actions is a crucial experience for infants in the 1st year of life (Reddy and Uithol, 2015). Unlike the free and spontaneous interactions caregivers may engage in with infants, routines are usually organized around a structured activity (Fantasia et al., 2014b). Routines provide infants with early opportunities to take part in a shared activity, and also allow them to become gradually more coordinated and collaborative with others. Previous research suggests that being involved in joint activities is critical for children’s development of memory (Sommerville and Hammond, 2007), planning and problem-solving skills (Radziszewska and Rogoff, 1988), and also more mature cooperative abilities (e.g., Brownell and Carriger, 1990; Ashley and Tomasello, 1998; Warneken et al., 2006, 2012).

However, being involved and participating in routine joint activities are two different aspects of interacting. For example, Henderson et al. (2013) have suggested that 10-month-olds understand collaborative goals of a shared activity only after having actively experienced that activity. Participating requires that a person assumes a more active role in an interaction. Do infants participate – in the sense of collaboratively engage – in shared routines? Research examining the development of collaboration and cooperative behaviors has primarily focused on children from 1 year of age, while evidence from developmental studies on infants’ early participation in joint, cooperative activities is scarce and controversial. Hubley and Trevarthen (1979, p. 58) were the first to define early mother–infant interactions as cooperative, in a way that “each of the subjects is taking account of the other’s interests and objectives in some relation to the extrapersonal context, and is acting to complement the other’s response”. They presented evidence of early cooperative understanding during early communicative interactions between young infants and their mothers, by showing that from 8 to 12 month infants increased their ability to integrate expressions of interpersonal communication with cooperative praxic acts (Hubley and Trevarthen, 1979). On the other hand, Keitel et al. (2014) cautiously proposed that infant perception of joint actions develops starting at 9 month and differs from their perception of individual actions; in other words, before 9 month they are not expected to cooperate.

One way to address the controversy about the development of shared intentionality and joint action (see Tollefsen and Dale, 2012, for a review) is to investigate infants’ contribution to the building up of a shared activity with others, by observing how they complement others’ actions with movements. That is, looking at infants’ motor behaviors during routines may shed light on infants’ awareness of others’ situated and goal-directed actions. As Smitsman and Corbetta (2010) have suggested, studying action development is fundamental to understanding how and what infants learn about their environment. Action anticipation (or prediction), for instance, has been extensively studied in the last two decades as a measure of infants’ developing understanding of the goals and intentions of others’ actions (Gredebäck and Melinder, 2010; Kanakogi and Itakura, 2010; see Hunnius and Bekkering, 2014, for a review). Moreover, Reddy et al. (2013) have suggested that infants’ anticipatory motor adjustments to being picked up may reveal their ability to understand and adjust to the incipient action by the mother in a participatory way. However, anticipating the other’s action in order to facilitate the onset of an activity is only part of the story. Supporting and dynamically coordinating with the other while the action unfolds also seems a crucial contribution for the action success.

Another way to explore whether infants have an understanding of a shared, familiar activity is to look at changes in infants’ responses to unexpected behaviors by the adult (e.g., infant’s reaction to maternal breach in engagement, or withdrawal from an ongoing interaction). Experimental paradigms based on such violations have been successfully used to investigate infants’ expectations in a range of different domains from very early on (Murray and Trevarthen, 1986; Baillargeon, 1994; Nadel et al., 1999). For example, previous research has shown that infants tend to look longer at their partner or shift their gaze frequently in response to unexpected behaviors (Phillips et al., 1992; Bertin and Striano, 2006). Looking at changes in infants’ behavior during a modified version of a routine activity may thus reveal infants’ expectations about or understanding of how that very activity should be performed, and consequently, tell us something about infant awareness of others’ intentions-in-action.

In the present study both these aspects – examining motor behaviors as means of complementing the other’s action, and observing behavioral changes in response to violations of a routine – have been used to look at infants’ contribution to being picked up.

### Being Picked Up

Previous research has shown that 4- to 5-month-old infants have expectations to be picked up when crying after waking up, showing signs of distress if the adult fails to do so (Lamb and Malkin, 1986). At around 6 to 7 months of age infants request to be picked up by lifting their arms up in response to mothers’ approach (Lock, 1984), although this response is strongly affected by the mother’s style of picking up and communication with the infant (Service, 1984). Recent evidence showed that being picked up also seems to involve a fair amount of postural and kinematic coordination by the infant. Reddy et al. (2013) found that when the caregivers’ approach was clear and visible, even 2-month-old infants made appropriate anticipatory adjustments to the mother’s pick-up action. Specifically, the authors found that infants increased the rigidity of their bodies, while general thrashing was reduced, and moved their extremities to create space for the mother to hold them comfortably, by widening or raising their arms. Interestingly, the authors also noticed a rotation of the head when infants were just about to be lifted, which may have served to increase stiffening in the neck muscles, thus reducing the lag of the head during the lift. This finding is in line with current literature on motor development showing that head control, already present around 3 months of age, is critical for a range of early behaviors, including those related to postural stability, motility and vision. In turn, this may support the argument about infants’ gradual involvement in social exchanges (for a review see Adolph et al., 2009).

### The Present Study

In light of these findings, in the present study we observed 3-month-old infants’ contribution to their mother’s movements over the entire duration of a pick-up episode when this was performed at a usual pace and with a delay before the lift. Reddy et al.’s (2013) study showed that at 2 months all anticipatory adjustments to approaching pick-up were in place, but the process was not yet as fluent as at 3 months. From 4 months infants began to be interested in the mother’s hands, which sometimes served to distract the infant from the pick-up itself; this distraction became very pronounced at 5 months and later. Thus, 3 months was the ideal age for studying infant responses to delays during a pick-up episode.

We chose to frame infants’ contribution in terms of their motor behavior, following preliminary observations of changes in their movements and limbs tension during a pilot study. A measure of Body Tension was thus created, and we hypothesized that this tension would increase over the course of a normal pick-up episode to reach its peak during lift. In contrast, we expected that infants’ Body Tension will decrease when the pick-up is delayed.

Following the observations by Reddy et al. (2013) on infants’ neck adjustment just before being lifted, we measured infants’ head sustain (i.e., Head Lag) as an additional measure of being prepared (or unprepared) to being lifted. If in a normal pick-up episode infants would keep their head in line with their body to sustain the mother’s lifting action, we hypothesized that a delay in the pick-up sequence would then leave infants unprepared to being lifted, showing a floppy head and thus a larger Head Leg.

Since our design involved a delay or violation from the usual experience infants have of the pick-up sequence, we also added two measures that have been extensively used in previous research on violation of expectations in infancy, namely shifts in gaze (Phillips et al., 1992) and affect displays (Legerstee and Markova, 2007).

## Materials and Methods

### Participants

Twenty-three 3-month-old infants (10 girls, *M*_age_ = 96.04 days, *SD* = 3.92 days) participated in the study. All infants were healthy at birth, Caucasian, and from lower to middle class families, as determined by parental reports on years of education. Maternal age at time of birth ranged from 26 to 37 years (*M*_age_ = 31 years, *SD* = 3.17 years). Volunteer parents were recruited through family centers, nurseries, and pre/antenatal classes in town. Ethical approval was obtained from the University Ethics Committee (University of Portsmouth) and informed consent was obtained from parents. Two dyads were excluded from the original sample of 25 infants due to the infants’ fussiness and lack of interest during the observations.

### Materials and Procedure

Mother–infant dyads were observed in a quiet, spacious room at a University Infant laboratory. Prior to the start of the testing session, mothers were asked whether their infants appeared to be showing any anticipation of their actions in general and, more specifically, of impending picks-up in various situations. Then the experimenter and the infant played for approximately 3–5 min to familiarize the infant with the new environment. The *Bayley Scales of Infant Development* – Second Edition (BSID-II; Bayley, 1993) were then administered to control for infants’ motor maturity, cognitive skills and equivalent developmental age. One infant scored lower than one percentile under the average on the Mental Scale (Mental Index score = 82). However, this infant’s behavioral responses were not different from the average responses of the other infants, thus this infant was included in the final sample. The BSID-II average assessment length was 12 min. Following the BSID-II assessment, infants were laid down on a mat (47 cm × 47 cm) placed on a low table (36 cm off the floor). Interactions were filmed with a digital camera that focused on the infant (recording at 30 frames per second).

All dyads were observed in two conditions: (1) Normal and (2) Delayed. In order to prevent changes in mothers’ usual pick-up routines, the normal pick-up always preceded the delayed pick-up. To observe a normal pick-up episode, mothers were instructed to chat with their infants and pick them up a few times during the interaction whenever they felt infants were comfortable and attentive, ensuring that the infants could see their arms as they approached to pick them up. Mothers attempted between two and four pick-up episodes overall. To choose one normal pick-up episode to be coded in this condition, three criteria were used by two independent judges to ensure their usability (see also Reddy et al., 2013): (i) the mother’s arms were approaching frontally and were therefore potentially visible to the infant; (ii) the infant’s gaze was directed toward the mother; and (iii) the episode was preceded by a period of engagement, increasing the likelihood of the infant wanting to be picked up. If more than one episode met these criteria, the first good episode was chosen. There was disagreement about the criteria in two cases, which was resolved following re-viewing of the video material.

To observe a delayed pick-up, mothers were asked to repeat the same procedure, but hold their hands on the infants’ waist for approximately 6 s before lifting. The end of the 6 s delay was signaled by the experimenter. Because our aim was to evaluate the effects of a breach in infants’ expectations, the Delayed condition was only observed once for each dyad. In one case, however, the mother had to repeat the delayed pick-up procedure due to the infant’s fussiness.

### Measures

#### Identifying Phases Within Pick-Up Episodes

Each normal and delayed pick-up episodes was divided into three phases: (1) Approach: beginning from the onset of the mother’s arms starting to approach the infant until Contact; (2) Contact: beginning from the onset of the mother’s hands contacting the infant’s waist until the onset of Lift; (3) Lift: beginning from the movements of mother’s hands on the infant’s waist until the infant’s body was completely detached from the mat.

One coder viewed and identified the frame points for the onset of Approach and Contact, and onset and offset of Lift for all infants in both conditions (Normal and Delayed). A second coder independently viewed 25% of the video material in both conditions. The coders disagreed on two pick-up episodes out of 24 (within 10 video frames, i.e., at 30 fps, 1/3 of a sec). Coefficients of agreement for each phase are presented in **Table 1**.

**Table 1:** Inter-Rater Reliability (calculated as Intra-Class Correlations; ICC) for all Measures used in the Present Study.

Measure		ICC
Phases	Approach	1
	Contact	0.999
	Lift	0.998
Body Tension		0.97
Gaze	Mother’s Face	0.967
	Mother’s Body	0.861
	Away from Mother	0.913
Affect	Positive	0.906
	Negative	1
Head Lag	Beginning Lift	0.996
	Midpoint Lift	0.998

Mean durations for each of these three phases were as follows: Approach = 2.49 s, Contact = 2.05 s and Lift = 1.54 s in the Normal condition, and Approach = 1.55 s, Contact = 8.32 s, and Lift = 1.48 s in the Delayed condition. As expected, the duration of the Contact phase was significantly longer for the Delayed than the Normal condition, *F*(1,22) = 195.93, *p* < 0.00, η^2^ = 0.899, 95% *CI* [5.34, 7.2], confirming that mothers were following our instructions. However, while there was no significant difference in the duration of the Lift phase between conditions (*p* = 0.566), the Approach phase was significantly longer in the normal compared to the delayed pick-up, *F*(1,22) = 5.279, *p* = 0.031, η^2^ = 0.194, 95% *CI* [0.09, 1.789]. The difference in Approach duration in the two conditions may be due the procedure order. Since the Delayed condition was always presented after at least one normal episode, mothers may have acquired familiarity with the procedure so that the delayed episode was generally quicker than the normal one(s).

#### Behavioral Coding

The following infant behaviors were coded in all three phases in both conditions: Body Tension, Head Lag, Gaze Shifts, and Positive and Negative Affect displays. The duration (relative to the duration of each phase in each condition for each infant) of *Body Tension* was measured as the onset and offset of simultaneous movements of arms and legs in any of the following combinations: *Arms* stretched out, widening out to the side, raising up, or stretching toward the mother; and *Legs* extending flat and raising slightly upward, or tucking up.

To assess infants’ stiffening of the neck when lifted, we measured their Head Lag during the Lift phase in both normal and delayed pick-ups. Using the video software Dartfish, we created this measure by calculating the angle between chin, chest and neck border for each infant at two points: (a) beginning of Lift, corresponding to the onset of the Lift phase, which was used as a baseline to control for each infant’s individual angle when the head was leaning on the mat; and (b) halfway through Lift, operationalized as the midpoint in time of the Lift phase, which was adjusted to account for individual variations in the Lift phase duration. If the infant’s head dropped backward while being lifted, then this resulted in an increase of the measured angle (i.e., decreased head-neck strength) at the midpoint of the Lift phase.

Infants’ *Gaze* was coded when directed to the mother’s *face*, the mother’s *body*, or *away* from the mother. We then measured how many times infants shifted their gaze from the mother’s face to away and from the mother’s face to the mother’s body during the Approach and Contact phases in both conditions.

Finally, the frequency of Positive and Negative Affect displays was coded and adjusted to the duration of each phase in each condition for each infant (i.e., frequency^∗^mean/actual duration of the phase). *Positive Affect* displays were defined as smiles (i.e., raised cheeks and corner of lips turned up with mouth open or closed) or laughs (i.e., raised cheeks, mouth open, lower, and upper gum visible, eyes open, or winked, possibly accompanied by some vocalizations), whereas *Negative Affect* displays were defined as frowns (i.e., furrowed brow and downturned mouth) or sad expressions (i.e., mouth, eye brows, and cheeks turned down) (see also Legerstee and Markova, 2007).

Infants’ behaviors were coded by one observer blind to the rationale of the study. Episodes were watched at least twice: initially at normal speed to identify relevant behaviors, and then frame by frame to identify onset and offset points of each behavior. A second observer (also blind to the rationale of the study) independently coded 25% of the video material in both conditions. Inter-observer reliability was assessed using the Intraclass Correlation Coefficient, and values ranged from 0.861 to 1 (see **Table 1**).

## Results

Means and standard deviations for all measures are presented in **Table 2**. Repeated-measures ANOVAs were computed separately for each infant behavior. Pairwise comparisons were adjusted with a Bonferroni correction.

**Table 2:** Descriptive Statistics for all Measures in Both Conditions and All Phases.

Measure	Normal		Delayed	
	*M*	*SD*	*M*	*SD*
**Body Tension (relative duration in ms)**				
Approach	0.39	0.30	0.46	0.34
Contact	0.61	0.22	0.40	0.17
Lift	0.65	0.33	0.17	0.18
**Head Lag (angle)**				
Beginning Lift	76.63	13.45	79.14	13.78
Midpoint Lift	89.08	13.92	105.43	12.78
**Positive Affect (relative frequency)**				
Approach	1.33	1.92	0.82	1.14
Contact	1.39	1.61	0.75	0.85
Lift	0.27	0.62	0.02	0.10
**Negative Affect (relative frequency)**				
Approach	0.19	0.79	0.35	1.13
Contact	0.01	0.06	0.76	0.83
Lift	0.16	0.46	0.40	0.58
**Gaze Shifts from Mothers’ Face to Away (relative frequency)**				
Approach	0.43	0.66	0.48	0.51
Contact	0.3	0.47	1.26	0.69
**Gaze Shifts from Mothers’ Face to Mothers’ Body (relative frequency)**				
Approach	0.26	0.45	0.74	0.69
Contact	0.35	0.49	1.18	0.89

**FIGURE 1:**
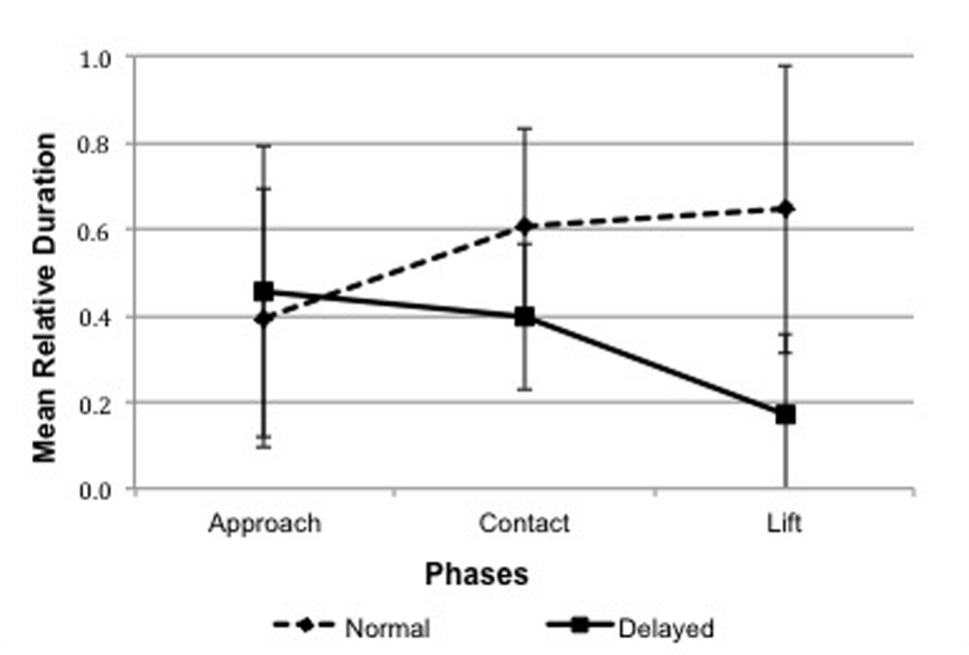
**Mean relative durations of Body Tension across the three phases in Normal and Delayed Pick-Up episodes**.

A repeated-measures ANOVA for Body Tension with condition (Normal, Delayed) and phase (Approach, Contact, Lift) as the within-subjects variables, showed a significant main effect of condition, *F*(1,22) = 24.48, *p* < 0.001, η^2^ = 0.527, 95% *CI* [0.120, 0.294], and a significant interaction between condition and phase, *F*(2,44) = 8.828, *p* = 0.001, η^2^ = 0.286. Simple contrasts revealed that the total duration (in terms of ms) of time were infants had their body tensed increased from Approach to Contact in the Normal condition (*p* = 0.016, 95% *CI* [-0.397, -0.035]), but decrease from Approach to Lift (*p* = 0.012, 95% *CI* [0.055, 0.513]) as well as from Contact to Lift (*p* = 0.004, 95% *CI* [0.065, 0.381]) in the Delayed condition (**Figure 1**).

**FIGURE 2:**
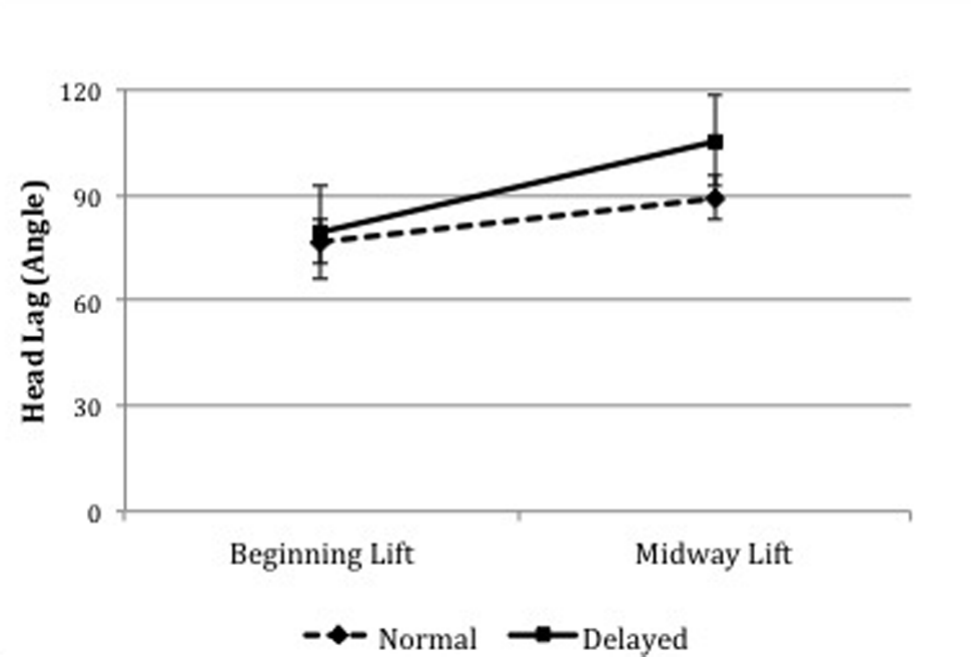
**Mean Head Lag angles at the beginning and midpoint of Lift in Normal and Delayed Pick-Up episodes**.

To compare Head Lag before and during Lift in the two conditions, a repeated-measures ANOVA was computed with condition (Normal, Delayed) and time (beginning lift, midway lift) as the within-subjects factors. Results revealed a significant main effect of condition, *F*(1,22) = 17.94, *p* < 0.001, η^2^ = 0.449, 95% *CI* [4.81, 14.04], and time, *F*(1,22) = 126.58, *p* < 0.001, η^2^ = 0.852, 95% *CI* [15.80, 22.94], as well as a significant interaction between condition and time, *F*(1,22) = 26.32, *p* < 0.001, η^2^ = 0.545 (**Figure 2**). While there was no difference between the conditions at the beginning of the Lift (*p* = 0.291, 95% *CI* [-7.32, 2.30]), simple contrasts showed that halfway through the Lift Head Lag was significantly higher in the delayed than in the normal pick-up, *F*(1,22) = 32.73, *p* < 0.001, η^2^ = 0.598, 95% *CI* [-22.28, -10.42], suggesting that infants’ neck had lost its tension and the head was not aligned with the rest of the body when the child was lifted after a delay.

A repeated-measures ANOVA on the frequency of Gaze Shifts with direction (face-to-mother’s body, and face-to-away), condition (Normal, Delayed), and phase (Approach, Contact) as the within-subjects factors, showed a significant main effect of condition, *F*(1,22) = 42.73, *p* < 0.001, η^2^ = 0.66, 95% *CI* [0.39, 0.76], and phase, *F*(1,22) = 15.39, *p* = 0.001, η^2^ = 0.412, 95% *CI* [0.14, 0.45], as well as a significant interaction between phase and condition, *F*(1,22) = 16.61, *p* = 0.001, η^2^ = 0.43. Simple contrasts indicated that in the Delayed condition Gaze Shifts were significantly more frequent during Contact than during Approach, *F*(1,22) = 37.66, *p* < 0.001, η^2^ = 0.631, 95% *CI* [-1.63, -0.81], while in the Normal condition there was no difference in Gaze Shifts between Approach and Contact (*p* = 0.852, 95% *CI* [-0.44, -0.52]). The direction of the shifts (i.e., from mother’s face to mother’s body vs. away) was not significant.

Finally, repeated-measures ANOVAs on affect displays with condition (Normal, Delayed) and phase (Approach, Contact, Lift) as the within-subjects variables, revealed a significant main effect of condition for Positive Affect, *F*(1,22) = 4.957, *p* = 0.037 η^2^ = 0.184, 95% *CI* [0.03, 0.89], and Negative Affect, *F*(1,22) = 6.583, *p* = 0.018, η^2^ = 0.23, 95% *CI* [0.66, 0.07], indicating a higher frequency of Positive Affect displays in the Normal (*M* = 0.996) than in the Delayed (*M* = 0.533) pick-up, and a higher frequency of Negative Affect displays in the Delayed (*M* = 0.504) than in the Normal (*M* = 0.122) pick-up. Moreover, there was a significant main effect of phase for Positive Affect, *F*(2,44) = 6.235, *p* = 0.004, η^2^ = 0.221, showing that, in both conditions, infants displayed significantly less positive affect during Lift (*M* = 0.148) compared to Approach (*M* = 1.073, *p* = 0.016, 95% *CI* [0.15, 1.71]) and Contact (*M* = 1.071, *p* < 0.001, 95% *CI* [0.43, 1.42]).

## Discussion

The goal of the present study was to provide evidence for the argument that cooperating with the caregiver’s action is embedded in the embodied participation in joint routines. To this end, we observed 3-month-old infants’ behaviors during a natural interaction, when mothers either picked up the infant normally or they delayed the pick-up sequence. Our results indicated that when the pick-up interaction unfolded normally infants tensed up their body, stiffened their neck (i.e., decreasing the lag between the chin and the chest) and displayed more positive affect than when the pick-up was delayed. In other words, when Contact was not followed by a lift within the usual time frame, infants released their arms and legs as well as their neck tension and displayed more negative affect.

We observed a typical constellation of gaze, affective displays and body movements, which varied in the two conditions. During Approach in both Normal and Delayed conditions, infants showed a tendency to look attentively at their mothers, smile or laugh, and thrust their legs or/and arms. When the pick-up sequence progressed normally, after Contact infants continued looking at their mothers – often maintaining their positive affect – and increased their body movements into a more regular pattern that was here coded as Body Tension. As the sequence turned into Lift, Body Tension peaked and most infants kept gazing at the mother, strengthening their neck with their head in a frontal position. Few infants turned their head sideways, which may represent another strategy to support their head to prevent a head lag, as suggested by Reddy et al. (2013). In contrast, when the pick-up was delayed after Contact, most of the infants began to display negative affects after approximately 3.5 s while mothers were keeping their hands on infants’ waist; some infants shifted their gaze back and forth from the mother’s face to her hands or away, and the majority of them decreased their body tension. These behavioral changes then continued during delayed Lift, where infants’ eye contact with their mothers continued to be fluctuating and they motor behavior weakened: the body tension dropped to the lowest point, and most of the infants manifested a loss of tension in the neck resulting in an increased head lag.

These results hold implications for our understanding of infants’ participation in shared actions that go beyond infants’ ability to adjust to or anticipate the mother’s action. Specifically, our findings suggest a particular sensitivity to the timing and sequence of the pick-up action as it unfolds, and possibly about the duration of each of its phases. Infants showed a similar motor response and gaze focus on the mother’s face during Approach in both conditions, which may be considered a “preliminary” phase signaling the beginning of the pick-up sequence. In the following phase, marked by the mothers’ contact with the infants’ waist, the increase in body tension and positive affect highlighted that infants gained most of their tension and then maintained it steadily throughout the lift. On the contrary, when the pick-up was delayed, infants lost their preparatory tension, indicating their sensitivity to the timing and sequence in which the pick-up action generally progressed – with the mother’s hands first on the waist and then moving down for lifting the infant’s up.

What does this suggest in terms of infants’ participation in a normal pick-up routine? Infants seem to invest their bodily and affective energy not only in anticipation to, but also contingently adjusting to the mother’s behavior during the entire unfolding of the action. The release of tension during the Delayed condition seems to support this argument. While an interpretation of infants’ participatory behaviors as either co-operative or based on simple associations remains to be addressed by future research, our findings indicate that infants supported and adjusted to their mothers’ timing of pick-up behaviors.

The increase of gaze shifts from Approach to Contact during the delayed pick-up, could be interpreted as an attempt to disambiguate the mother’s behavior, as previous research has reported (Phillips et al., 1992; Behne et al., 2005). Yet, since these shifts were equally distributed between gaze away and to the mother’s body, it is difficult to specify their exact function. Most mothers did not show any affective expressions during the delay of the pick-up, while few of them smiled or vocalized to the infant when she or he looked at them. One possible explanation could be that infants disengaged from the interaction to avoid distress, as suggested by studies using the Still-Face Face paradigms (Tronick et al., 1978; Adamson and Frick, 2003). Alternatively, gaze shifts may be an attempt to grasp and share the mother’s attention in an ambiguous situation (Amano et al., 2004), and thus allow infants to track their mothers’ action and try to make sense of it.

Some limitations of the present study need to be addressed in future research. First, the fact that the Normal pick-up was always performed first may have influenced infants’ responses to the subsequent delay in the pick-up sequence by, for example, increasing the infants’ attention to the violation of the usual way they are picked up; counterbalancing the two conditions may have helped to have a clearer effect of the delay on the infant’s behavior. At the same time, asking mothers to introduce a delay in their natural pick-up routine before picking up their infants may have disturbed their naturally occurring behaviors. Future studies exploring the pick-up routine need to consider these two aspects and their implications seriously. Second, being picked up twice within a relatively short period of time might have overstretched infants’ attentiveness, resulting in the overall decrease of participation showed by infants in the delayed pick-up episode. However, our results indicate that infant behaviors were comparable in the approach phase of both conditions, and only during contact did the infants begin to realize that ‘something is not quite right’. Despite these findings, our study design did not allow us to determine the precise point in which infants detected the violation in the pick-up flow and changed their behavior. This is problematic, conceptually as well as practically, because infants could make allowances for the delay by expecting to be picked up for some time and thus behaving as if the pick-up was not delayed. Yet, it could be argued that by analyzing the whole contact phase, where the change occurred, and not the specific time where the infant would have normally been picked up until it eventually was, we accounted for these individual allowances, and thus consider this a conservative approach. Finally, being picked up was de-contextualized and not related to any previous activity nor functional to the following one, as is usually the case. This may have affected the infants’ natural behavior. Future research aiming to investigate infants’ participation in daily, familiar practices (not only a pick-up routine) would strongly benefit from observing mothers and infants interacting in their natural environment, such as at home. We believe that such a change in setting may reveal aspects of infants’ participation as rich and functional, which cannot be observed in other, more artificial contexts.

## Conclusion

Our study suggests that, when being picked up, infants are not passive recipients of actions performed on them, but alert and active participants behaving according to the emergent features of the activity. Early signs of co-operative participation can be found in the way infants supported and responded to their mothers’ timing of movements, facilitating or adjusting to the pick-up action as it unfolded. This is in line with a more dynamic and developmental approach to the study of cooperation that takes into account the role of infants’ daily experience with shared practices (see also Fantasia et al., 2014a). Indeed, by participating in early routines infants take part in a process of “conventionalization” of social practices, which integrates affective, cognitive, communicative and kinetic aspects. What makes behaviors predictable for infants may lie in the experience of moving together, lived through a multiplicity of sensory modality, including proprioception. As Fogel and Thelen (1987) have proposed, social behavior is not behavior *toward*, but mostly behavior *with* others. Of course, should an adult decide to pick-up an infant against her or his will, she would easily succeed without much effort. Yet, the motivation and pleasure achieved through a pick-up interaction might probably not be the same, as infant responsiveness and engagement during the pick-up is arguably crucial in its potential for motivating the caregiver and fostering the intersubjective exchange.

## Author Contributions

VR has made a substantial contribution during the preliminary phases of the study conception, design, and theoretical building up of the arguments. She also made an important contribution to the interpretation of data.

## Conflict of Interest Statement

The authors declare that the research was conducted in the absence of any commercial or financial relationships that could be construed as a potential conflict of interest.
